# Assessing the consistency and sensitivity of the neural correlates of narrative stimuli using functional near-infrared spectroscopy

**DOI:** 10.1162/imag_a_00331

**Published:** 2024-10-24

**Authors:** Matthew Kolisnyk, Sergio Novi, Androu Abdalmalak, Reza Moulavi Ardakani, Karnig Kazazian, Geoffrey Laforge, Derek B. Debicki, Adrian M. Owen

**Affiliations:** Graduate Program in Neuroscience, Schulich School of Medicine and Dentistry, Western University, London, ON, Canada; Western Institute for Neuroscience, Western University, London, ON, Canada; Department of Physiology and Pharmacology, Western University, London, ON, Canada; Clinical Neurological Sciences, Western University, London, ON, Canada; Department of Psychology, Western University, London, ON, Canada

**Keywords:** functional near-infrared spectroscopy, movie watching, movie listening, higher-order cognition, inter-participant reproducibility, inter-subject correlation, machine learning

## Abstract

Investigating how the brain responds to rich and complex narratives, such as engaging movies, has helped researchers study higher-order cognition in “real-world” scenarios. These neural correlates are particularly useful in populations where behavioral evidence of cognition alone is inadequate, such as children and certain patient populations. While this research has been primarily conducted in fMRI and EEG, whether functional near-infrared spectroscopy (fNIRS) can reliably detect these neural correlates at an individual level, which is required for effective use in these populations, has yet to be established. This study replicated widespread inter-subject correlations (ISCs) in the frontal, parietal, and temporal cortices in fNIRS in healthy participants when they watched part of the TV episode*Bang! You're Dead*and listened to an audio clip from the movie*Taken.*Conversely, these ISCs were primarily restricted to temporal cortices when participants viewed scrambled versions of those clips. To assess whether these results were reliable at the single-participant level, two follow-up analyses were conducted. First, the consistency analysis compared each participant’s ISCs against group results that excluded that individual. This approach found that 24 out of 26 participants in*Bang! You’re Dead*and 20/26 participants in*Taken*were statistically similar to the group. Second, the sensitivity analysis measured whether machine-learning algorithms could decode between intact conditions and their scrambled counterparts. This approach yielded balanced accuracy scores of 81% in*Bang! You’re Dead*and 79% in*Taken*. Overall, the neural correlates of narrative stimuli, as assessed by fNIRS, are reproducible across participants, supporting its broad application to clinical and developmental populations.

## 1 Introduction

Recent studies have been increasingly using naturalistic paradigms—experimental tasks designed to emulate the experience of everyday life—to capture the underlying neural signature of complex cognition (Hasson et al., 2008;Sonkusare et al., 2019;Spiers & Maguire, 2007;Vanderwal et al., 2019). One such naturalistic paradigm is movie-watching and listening (Bartels & Zeki, 2004;Hasson et al., 2004). Movies are unique experimental stimuli because they enable the exploration of a variety of higher-order cognitive processes, such as language comprehension, attention, memory, plot following, and feelings of suspense (Chen et al., 2017;Hasson et al., 2008;Naci et al., 2014;Nguyen et al., 2019). Moreover, these higher-order cognitive processes produce reliable neural correlates when measured with functional magnetic resonance imaging (Gao et al., 2020;Hasson et al., 2010;Hlinka et al., 2022;Naci et al., 2014;Vanderwal et al., 2019;J. Wang et al., 2017; fMRI). These factors have prompted the widespread use of movies in psychological, neuroscientific, and clinical research (Alexander et al., 2017;Eickhoff et al., 2020;Hasson et al., 2008;Nastase et al., 2021).

Movie narratives are particularly appropriate in populations where it is challenging or otherwise impossible to detect higher-order cognitive functioning behaviorally (Alexander et al., 2017;Naci et al., 2017). This includes developmental and pediatric populations which use the neural correlates of movie narratives to measure cognitive capacities in children and estimate their association with mental health, developmental, and learning disorders (Alexander et al., 2017;Lyons et al., 2020;Richardson et al., 2018;Vanderwal et al., 2019) as well as neurodegenerative and critically brain-injured patients where movie narratives are used to assess for evidence of higher-order cognitive capacities (Huntley et al., 2023;Laforge et al., 2020;Naci et al., 2014). In all cases, observing neural activity in response to movie narratives provides a basis for attributing higher-order cognitive capacities to these groups. Thus, these attributions enable a better understanding of these populations as well as the potential to inform clinical decision making. Given these possibilities, it is essential that the neural correlates that are the basis of these judgments are robust and reproducible at a single-participant level.

Currently, the gold standard imaging modality for inter-participant reproducibility is fMRI. For example, inNaci et al. (2014), synchronization in neural activity in the frontoparietal network was observed in all 12 controls when they watched an edited version of the Alfred Hitchcock TV episode*Bang! You’re Dead*. The protocol was extended to two critically brain-injured patients. Due to their injuries, these patients’ behavioral responses to commands were unreliable or absent, making it unclear whether they had any residual cognitive capacities. Remarkably, the researchers found that one patient’s frontoparietal activity was significantly synchronized with that of controls, suggesting that the patient was experiencing the movie similarly to the healthy participants. Despite the successful applications of fMRI, it has drawbacks in that it is relatively expensive, challenging to administer, and may be unsuitable for many naturalistic paradigms.

Functional near-infrared spectroscopy (fNIRS) appears to be a suitable alternative for scenarios in which fMRI is not viable or ideal (Ayaz et al., 2022;Scholkmann et al., 2014). Owing to its main features, such as affordability, portability, and versatility, fNIRS holds great potential to be applied to pediatric and neonatal (Peng & Hou, 2021;Tang et al., 2024), neurodegenerative (Blum et al., 2022;Keles et al., 2022;Kuruvilla et al., 2013), and critically brain-injured (Abdalmalak et al., 2020;Bicciato et al., 2022;Shu et al., 2023;L. Wang et al., 2022) populations. Given these advantages, fNIRS has also been increasingly used to measure neural activity in naturalistic contexts (seePinti et al., 2018;Quaresima & Ferrari, 2019for a review), ranging from social interaction (Hakuno et al., 2018;Hirsch et al., 2018), real-world memory studies (Burgess et al., 2022;Pinti et al., 2015), as well as narrative stimuli (Fishell et al., 2019;Mizrahi & Axelrod, 2023;Rowland et al., 2018;Somech et al., 2022). By shining and detecting near-infrared light on the surface of the scalp, fNIRS estimates oxy-(HbO) and deoxy-hemoglobin (HbR) from the cortex, relying on neurovascular coupling to infer brain activity (Boas et al., 2014;Ferrari & Quaresima, 2012;Huppert, 2006;Jöbsis, 1977;Scholkmann et al., 2014;Steinbrink et al., 2006). Despite this increased interest in the use of fNIRS in clinical settings (Abdalmalak et al., 2017,^2021^;Amyot et al., 2019;Kazazian et al., 2021;Kempny et al., 2016;Quiroga et al., 2022;Rupawala et al., 2018) and with naturalistic stimuli (Mizrahi & Axelrod, 2023;Pinti et al., 2018;Quaresima & Ferrari, 2019;Somech et al., 2022), the reproducibility and sensitivity of fNIRS to detect neural activation at the individual level is understudied (Novi, Forero, et al., 2020).

In this work, healthy participants watched a clip from the TV episode*Bang! You’re Dead*and listened to a clip from the movie*Taken*while monitored with fNIRS. In addition to these movie clips, participants watched or listened to scrambled versions of those clips. We hypothesized that both intact clips would lead to higher levels of synchronization compared to their scrambled counterparts, particularly in frontal and parietal regions known to be involved in higher-order cognitive processing of movie narratives (Laforge et al., 2020;Naci et al., 2014). Moreover, we predicted that frontal and parietal activity would be predicted by a measure of qualitative experience—the feelings of suspense felt during the clip—further establishing the relation between these regions and higher-order cognitive processing. Next, we measured the single-participant reproducibility of fNIRS by computing two separate measures: consistency and sensitivity. Consistency was measured as the similarity of a given participant to the group results that exclude that participant, whereas sensitivity was measured by whether machine-learning algorithms could decode between intact and scrambled narrative stimuli for each participant. Our results indicate that fNIRS can be used to extract consistent and sensitive synchronization patterns at the individual level, implicating fNIRS as a promising tool to reliably measure the neural correlates of movie narratives.

## 2 Methods

### 2.1 Participants

Thirty participants (*Mean age*= 23.3,*SD*= 3.1,*Range*= 19-31; 17 females) from Western University participated in this study. All participants were compensated for their time ($20/hour). Four participants were excluded from the analysis (one due to data acquisition issues and three rejected for having no good quality short channels), resulting in 26 participants being included in the subsequent analyses. All participants provided written informed consent before beginning the study. The study was approved by the Research Ethics Board at Western University in compliance with the Tri-Council Policy Statement (TCPS): Ethical Conduct for Research Involving Humans guidelines.

### 2.2 Stimuli

Participants were presented with four narrative stimuli (two*Intact*and two*Scrambled*). One*Intact*audiovisual TV clip (*Bang! You’re Dead!*; hereby referred to as*BYD*) and its*Scrambled*counterpart (hereby referred to as*BYD Scrambled*) and one*Intact*audio movie clip (*Taken*) and its*Scrambled*counterpart (*Taken Scrambled*). The*BYD*stimulus is an edited and shortened black-and-white TV episode from “Alfred Hitchcock Presents — Bang! You’re Dead” (seeLaforge et al., 2020;Naci et al., 2014for details). In short,*BYD*depicts a scenario involving a boy who finds his uncle’s gun, believing it to be a toy. The story’s suspense builds as he proceeds to load and point the gun at several individuals, eventually culminating with him discharging the weapon.*BYD Scrambled*is identical to*BYD*, but one-second increments of the movie were pseudo-randomly re-ordered, producing a coherent visual scene—one that is effectively identical to the unedited version—but without narrative structure. The*Taken*stimulus has also been described in detail elsewhere (Laforge et al., 2020;Naci et al., 2017). It is an audio excerpt from the movie “Taken”.*Taken*depicts an initially tense phone call between a father and daughter that begins with the father upset that his daughter is attending a trip abroad without following his rules. The tension escalates when kidnappers break into her hotel and capture her. The phone call ends with the father vowing to find the kidnappers.*Taken Scrambled*is effectively identical to*Taken*but with its spectral properties rotated, which distorts the higher-order audio features (e.g., speech) but maintains the temporal structure of the sound and lower-level audio features.*BYD*,*BYD Scrambled*,*Taken*, and*Taken Scrambled*lasted 478, 463, 312, and 302 seconds, respectively.

### 2.3 Procedure

Participants were presented with four stimuli: two audiovisual and two audio-only. For the audiovisual stimuli, participants were instructed to view and listen to the stimuli as they would typically do while watching a movie. For the audio-only stimuli, participants were instructed to look at a fixation cross presented in the middle of a computer screen while listening to the stimuli. Each stimulus was separated by 30 seconds of rest, during which participants were asked to maintain fixation and remain still with no audiovisual information.*Intact*stimuli always followed their*Scrambled*counterpart to avoid the possibility of prior knowledge of the plot from carrying over to their*Scrambled*versions. Moreover, the order of presenting the audiovisual or audio-only stimuli was counterbalanced across participants to account for covariates such as cognitive fatigue or decreases in fNIRS signal quality across the experiment. Each participant was given a self-paced break between the audiovisual and audio-only stimuli, if needed. SeeFigure 1Afor an overview of the experimental procedure.

**Fig. 1.**
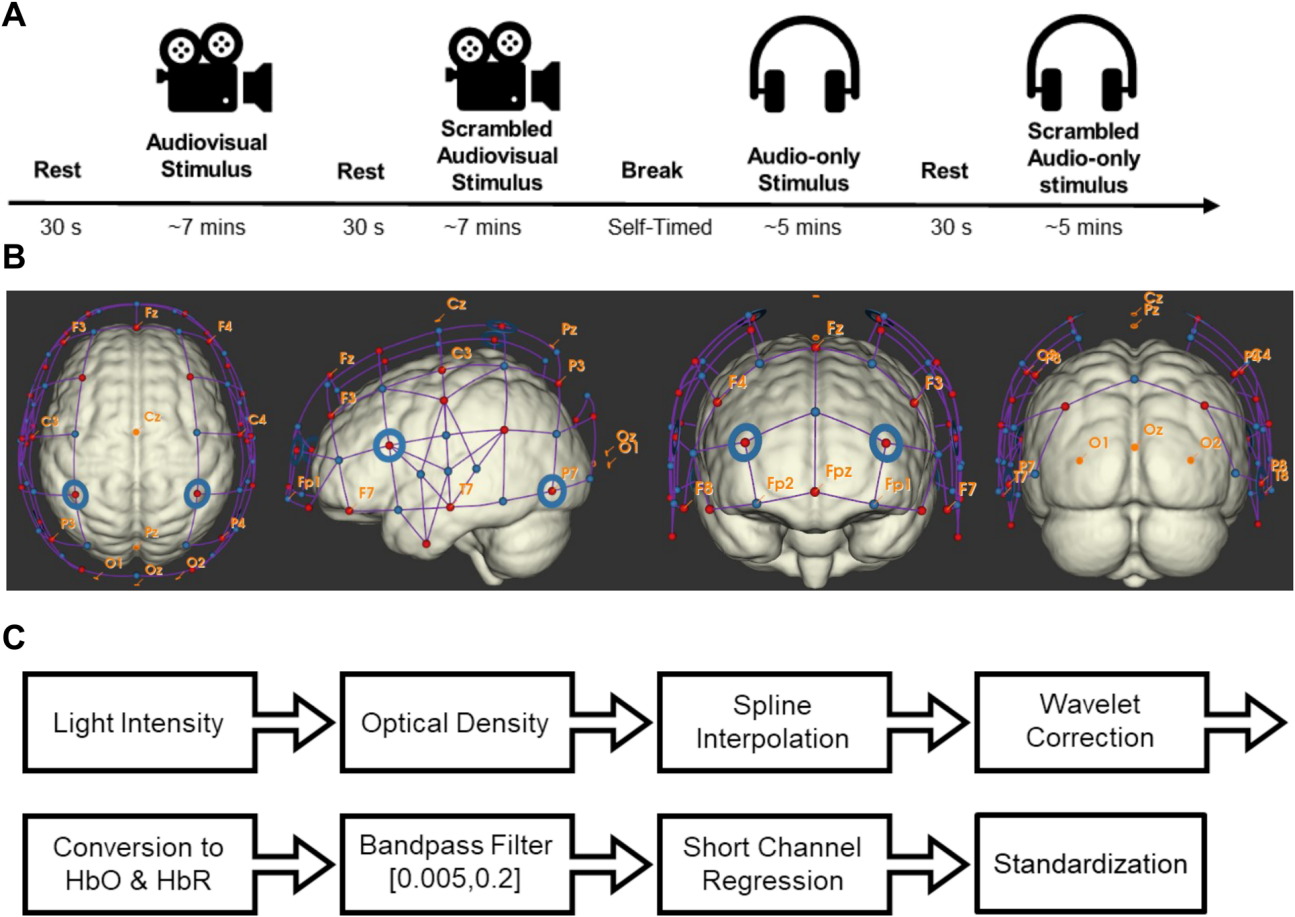
(A) Diagram showing the procedure of the experiment, which includes two audiovisual stimuli (from the TV episode “Alfred Hitchcock Presents — Bang! You’re Dead” (1961) and two audio-only clips (from the movie “Taken” (2008)). One of the audiovisual and audio-only stimuli were unadjusted (which we referred to as*Intact*), and the remaining two stimuli were manipulated to prevent following the narrative’s plot (which we referred to as*Scrambled*). The audiovisual*Scrambled*clip had each one-second increments of the movie pseudo-randomly re-ordered, producing a coherent visual scene that lacked a narrative structure. The audio-only scrambled clip was the result of a spectral rotation which rendered speech indecipherable but maintained the basic auditory properties of the stimuli. Note whether the audio-only clips or the audiovisual clips were shown first were counterbalanced, whereas*Scrambled*clips always preceded their*Intact*counterpart. (B) Four model brain images show the placement of sources (red dots) and detectors (blue dots) for horizontal, lateral, frontal, and posterior views (generated with NIRSite version 2023.9). (C) FNIRS data preprocessing pipeline.

### 2.4 Data acquisition

FNIRS data were acquired with a continuous-wave system (NIRScoutXP, NIRx Medical Systems; Berlin, Germany) at 3.9063 Hz with 32 sources and 39 detectors, allowing 121 regular (i.e., source-detector distance around 3 cm) and 8 short channels (i.e., source-detector distance of 0.8 cm). Data from 16 participants were collected with four wavelengths (lasers centred at 785, 808, 830, and 850 nm) and 14 with two wavelengths (LEDs centred at 760 and 850 nm). The probe covered most of the head, including the frontal, temporal, parietal, and a small part of the occipital lobe (seeFig. 1B). The sources and detectors were placed onto the participant’s head with a standard 10-20 cap from EasyCap (Brain Vision).

### 2.5 fNIRS data analysis

#### 2.5.1 Preprocessing

All preprocessing was conducted using custom scripts in MATLAB (Mathworks Inc; Version 2020b) based on HOMER 2 functions (Huppert et al., 2009). Light intensity was converted to optical density and then corrected for motion artifacts with a hybrid algorithm that employs spline interpolation followed by wavelet decomposition (Di Lorenzo et al., 2019;Novi, Roberts, et al., 2020; see theSupplementary Materialsfor information about the proportion of motion artifacts in the data). The cleaned optical density data were converted into oxy-(HbO) and deoxy- hemoglobin (HbR) via the modified Beer-Lambert law (Delpy et al., 1988):

$$\left\lceil \begin{array}{c} \Delta HbO\\ \Delta HBR \end{array}\right\rceil ={\left[\begin{array}{cc} \varepsilon_{HbO}^{\lambda_{1}} & \varepsilon_{HbR}^{\lambda_{1}}\\ \varepsilon_{HbO}^{\lambda_{2}} & \varepsilon_{HbR}^{\lambda_{2}} \end{array}\right]}^{-1}\left[\begin{array}{c} \frac{\Delta OD\left(\Delta t,\lambda_{1}\right)}{DPF\left(\lambda_{1}\right)\rho }\\ \frac{\Delta OD\left(\Delta t,\lambda_{2}\right)}{DPF\left(\lambda_{2}\right)\rho } \end{array}\right]$$

Whereεis the extinction coefficient, which is a constant reflecting how strongly hemoglobin absorbs light at a given wavelengthλ$(\varepsilon_{HbO}^{760}=645cm^{-1}/\left(\frac{moles}{liter}\right)$,$\varepsilon_{Hb}^{760}=$$1669cm^{-1}/\left(\frac{moles}{liter}\right)$,$\varepsilon_{HbO}^{785}=787cm^{-1}/\left(\frac{moles}{liter}\right)$,$\varepsilon_{Hb}^{785}=996.65cm^{-1}/\left(\frac{moles}{liter}\right)$,$\varepsilon_{HbO}^{808}=903cm^{-1}/\left(\frac{moles}{liter}\right)$,*$\varepsilon_{Hb}^{808}=804cm^{-1}/\left(\frac{moles}{liter}\right)$*,*$\varepsilon_{HbO}^{830}=1008cm^{-1}/\left(\frac{moles}{liter}\right)$*,*$\varepsilon_{Hb}^{830}=778cm^{-1}/\left(\frac{moles}{liter}\right)$,$\varepsilon_{HbO}^{850}=1097cm^{-1}/\left(\frac{moles}{liter}\right)$*,*$\varepsilon_{Hb}^{850}=781cm^{-1}/\left(\frac{moles}{liter}\right))$*, where these values were acquired using the*GetExtinctions*function from HOMER 2.ΔODindicates the observed change in optical density at a given wavelengthλacross some timet.DPFis the differential pathlength factor, which accounts for the increased length light may travel based on the scattering properties of tissue at a given wavelengthλ, andρis the Euclidean distance between a source and detector that define a channel. The DPF was computed for each wavelength and participant ([$DPF^{760}:Mean=6.060,SD=0.095;DPF^{785}:$$Mean=6.045,SD=0.073;DPF^{808}:Mean=5.849,SD=0.073;$$DPF^{830}:Mean=5.521,SD=0.073;DPF^{850}:Mean=5.037,$SD=0.091]) using a method which accounts for small variations due to the age of each participant (Scholkmann & Wolf, 2013). HbO and HbR signals were band-pass filtered between 0.005 and 0.20 Hz with a Butterworth filter. Next, scalp hemodynamics were regressed using short channel regression (Brigadoi & Cooper, 2015;Saager & Berger., 2005). Specifically, HbO and HbR measurements from good-quality short channels were regressed from each long channel. A short channel was defined as being of high quality if there was a visible power spectrum peak at ~1 Hz in the raw light intensity data in each wavelength. Principal Component Analysis (PCA) was applied to all clean HbO and HbR short channels (using the Singular Value Decomposition algorithm) prior to regression to avoid convergence problems due to collinearity between short channels (Abdalmalak et al., 2022;Santosa et al., 2020;Wyser et al., 2022). All principal components were included as regressors. Last, each cleaned hemoglobin time series was standardized by subtracting its mean and dividing by its standard deviation. The preprocessing pipeline is depicted inFigure 1C.

#### 2.5.2 Inter-subject correlation

Inter-subject correlation (ISC) analysis was used to determine neural synchronization across participants during movie watching and listening (Hasson et al., 2004;Nastase et al., 2019). Specifically, ISCs were computed by calculating the Pearson correlation coefficient between the hemoglobin time series of a given participant and the averaged time series across all remaining participants for each channel, stimulus, and hemoglobin independently. Next, we applied the Fisher transformation and averaged the correlation values of HbO and HbR for each channel, resulting in 121 ISCs (one per regular channel) per left out participant per stimulus condition (the procedure was repeated so that every participant was “left out” one time). Note that the HbO and HbR ISCs were averaged to produce a robust measure of ISC, as increases in HbO are expected to produce decreases in HbR, resulting in ISCs that are expected to be similar.

#### 2.5.3 Group analysis

Group-level statistical analyses were performed on ISCs from each condition (*BYD*,*BYD Scrambled*,*Taken*,*Taken Scrambled*) and for two between-condition comparisons (*BYD*>*BYD Scrambled*and*Taken*>*Taken Scrambled*). No comparisons were made between the audiovisual and audio-only stimuli, with the exception of ((*BYD*>*BYD Scrambled*) vs. (*Taken*>*Taken Scrambled*)), which can be found in the*Exploring Neural Differences in Higher-Order Cognitive Processes Between BYD and Taken*section in theSupplementary Materials. Statistical significance was determined using a permutation testing approach. Specifically, 1000 phase-scrambled versions of the original data (i.e., surrogates) were generated to derive null hypotheses for statistical testing. To construct the phase-scrambled surrogates, the Fourier transform was applied for each hemoglobin time series for each channel. The phase was then rotated by adding a random value between 0 and 2π, while the amplitude of the signal was held constant. The phase randomization was the same for both HbO and HbR to preserve the expected negative correlation between HbO and HbR. Importantly, these phase-scrambled surrogates maintain the structure and, critically in the case of fNIRS, the autocorrelation of the original signal, enabling accurate comparison with the original signal (seePrichard & Theiler, 1994;Regev et al., 2013for additional details).

The phase-scrambled surrogates were used to compute the distributions of ISCs values obtainable by chance. FDR correction was applied to these distributions using the max*t*method (Nichols & Holmes, 2002). For each condition, a one-tailed group-level*t*-test was conducted on ISCs derived from the phase-scrambled surrogates. The maximum*t*-scores obtained across channels were recorded for each surrogate, which generated the null hypothesis for their respective condition. The identical procedure was used for the two between-condition comparisons; only the ISCs from the*Scrambled*conditions were subtracted from the*Intact*conditions prior to running the one-tailed*t*-test. This procedure was completely effective at controlling for spurious correlations in the data, as verified on an independent resting state dataset (Abdalmalak et al., 2022; see*Validation of Inter-subject Correlation Analysis Using Resting State Data*in theSupplementary Materials).

#### 2.5.4 Suspense analysis

To substantiate that the neural differences between*Intact*and*Scrambled*conditions reflected higher-order processing of the movie’s narrative, suspense ratings were used to predict the group averaged HbO and HbR time series. These suspense ratings, reflecting a scale from 1 (minimal suspense) to 10 (peak suspense), were obtained at approximately two-second intervals from an independent cohort of 20 participants (Laforge et al., 2020;Naci et al., 2014) while they watched*BYD*or listened to*Taken*. Consistent with previous fMRI work (Naci et al., 2014), group-averaged suspense ratings were used as a regressor within a general linear model (GLM) to predict group-averaged HbO and HbR time series for both the*BYD*and*Taken*condition. Unlike previous studies, the respective*Scrambled*hemoglobin time series was added to the GLM model as a nuisance regressor. The additional nuisance regressor aimed to remove spurious correlations due to temporally dynamic patterns of the hemoglobin time series that may be correlated with the suspense ratings only by chance (e.g., changes in audio volume, auditory envelope). HbO and HbR time series were downsampled to match the same frequency of the suspense ratings. The*p*-values of the betas were FDR corrected via the Benjamini-Hochberg method, where*q*< 0.05 denoted significance (Benjamini & Hochberg, 1995).

#### 2.5.5 Inter-participant reproducibility

##### 2.5.5.1 Leave-one-out (LOO) datasets

To accurately estimate inter-participant reproducibility within a dataset, each individual participant’s data had to be compared with data that was independent of their own. To this end, a selected participant’s data were omitted from the dataset, and ISCs were recalculated on the remaining participants (identically as described in thesection 2.5.2). This procedure was repeated for each participant, generating 26 unique datasets in total. For instance, in the first leave-one-out (LOO) dataset, ISCs were computed using data from participants 2 through 26. This dataset would then be used to estimate the reproducibility of participant 1’s ISCs. In essence, each left-out participant is treated like a newly acquired “patient”, where their actual ISCs are compared to a “healthy control” dataset.

##### 2.5.5.2 Masking

To derive the mask of channels used to measure inter-participant reproducibility, the group analyses were recomputed on each LOO dataset. Specifically, this was done for each experimental condition (*BYD*,*BYD Scrambled*,*Taken*,*Taken Scrambled*) as well as for the two between condition comparisons (*BYD*>*BYD Scrambled*, and*Taken*>*Taken Scrambled*). This approach yielded 26 different masks per comparison. This approach avoids potential bias and double dipping as the mask used to measure inter-participant reproducibility was constructed on data that did not include the participant whose reproducibility was being tested.

##### 2.5.5.3 Consistency analysis

The consistency of a given participant’s ISCs was estimated by calculating a similarity score between the participant and their corresponding LOO dataset. Specifically, the normalized dot product was computed between the*Intact*ISCs of a left-out participant (as computed insection 2.5.2) and the group-averaged*Intact*ISCs computed from the remaining participants. This calculation only used channels from the*Intact*>*Scrambled*masks (e.g.,*BYD*>*BYD Scrambled*or*Taken*>*Taken Scrambled*), which were derived from the masking procedure described above. These channels were used as they were expected to isolate for higher-order cognitive processing specific to each narrative. The dot product was normalized for each participant to account for the different number of significant channels within each mask. Formally, this calculation is as follows:

$$\frac{1}{n}\sum_{i=1}^{n}a_{l,i}g_{l,i}$$

Where$g_{l,i}$is a vector of averaged ISC values across participants from the left-out datasetland$a_{l,i}$is a vector of ISCs from participantl, and*n*is the size of the mask. The normalized dot product was used over other similarity scores because it preserves both the magnitude and the direction of the similarity. Both magnitude and direction are important as only the participants who show systematically*lower*ISCs compared to the rest of the group are of interest as that would call into question the reproducibility of their ISCs.

To validate this approach, two verification steps were implemented. First, the identical procedure to above was applied instead using a given participant’s*Scrambled*ISCs rather than their*Intact*ISCs. That is, the normalized dot product was computed between the*Scrambled*ISCs and the group averaged ISCs from the*Intact*condition using the respective*Intact*>*Scrambled*mask. Then, a two-sample*t*-test was used to compare the normalized dot product computed from*Scrambled*conditions to those computed from the*Intact*condition, where*p*< 0.05 denotes a significant result. Second, the normalized dot product was recomputed using ISCs derived from phase-scrambled time series. Specifically, a set of 1000 phase-scrambled time series was computed for each LOO dataset. A null distribution of normalized dot products, representing chance level similarity, was then used to compare a participant’s actual normalized dot product to what is possible by chance. A*p*value was computed for each participant which reflected the number of times the actual normalized dot product was less than the normalized dot products computed on the phase-scrambled data. To adjust for multiple comparisons across participants, FDR correction was applied via the Benjamini-Hochberg method, with*q*< 0.05 denoting significance (Benjamini & Hochberg, 1995).

##### 2.5.5.4 Sensitivity analysis

To assess the sensitivity of single-participant ISCs, a machine-learning approach was used to determine whether ISCs could be used to decode between (1)*BYD*and*BYD Scrambled*and (2)*Taken*and*Taken Scrambled*conditions. To obtain a representative and robust estimate of performance, 17 machine-learning classifiers were used (available via sci-kit learn version 0.23.1;Pedregosa et al., 2011). The classifiers were selected to obtain a diverse range of existing classification approaches such as the tree-based methods (extra trees and bagging) as well as their gradient boosting extensions (Adaboost and XGBoost), linear models (logistic and ridge), support vector machines (SVM and nu-SVM), Bayesian (naïve Bernoulli and Gaussian), Gaussian (Gaussian process), semi-supervised (label propagation and spreading), discriminant analysis (linear and quadratic discriminant), and nearest neighbor (K-nearest neighbor, nearest centroid).

Each classifier was trained independently 26 times (once for each LOO dataset). Hyperparameter tuning was performed using the hyperopt package (version 0.2.7;Bergstra et al., 2013), with the objective to maximize the median balanced accuracy score obtained from threefold cross-validation on the training data. Hyperopt uses the Tree-structured Parzen Estimator (TPE) method, which is a Bayesian hyperparameter optimization framework. Specific details about which training and hyperparameter tuning can be found in theTable 5Sin the Supplementary Materials and code available online. Once optimized, each classifier was tested on the original ISCs of the participant (as computed insection 2.5.2) who was omitted from the current LOO dataset. The training and testing datasets were masked by channels in their respective LOO mask that were significant in either of the*Intact*or*Scrambled*conditions.

The classifiers’ predictions were pooled to determine whether the test ISCs were predicted to be from the*Intact*or*Scrambled*conditions. This polling procedure is otherwise known as “voting”, where the results of an ensemble of classifiers are aggregated to form a final, robust prediction (Breiman, 1996;Kuncheva & Whitaker, 2003). A given classifier’s vote was proportional to their optimized balanced accuracy score on the training set. The overall performance of this final prediction was measured by the balanced accuracy obtained on the test set.

The performance of the model was statistically evaluated by comparing the actual balanced accuracy to a null distribution of balanced accuracy scores derived from permutation testing. The identical approach to above was conducted, and only the true condition labels were shuffled. This was done for 1000 iterations with different shuffled labels to generate a null distribution of balanced accuracy scores. This same procedure was applied to determine significant recall (True*Intact*/ (True*Intact*+ False*Scrambled*)) and precision (True*Intact*/ (True*Intact+*False*Intact*)) scores.

Last, to explore which channels were driving the decoding performance, the identical machine learning approach was applied at the single-channel level. Specifically, for both*BYD*vs.*BYD Scrambled*and*Taken*vs.*Taken Scrambled*, ISCs from a single channel were used as a sole feature for the machine-learning approach to be trained and tested on. This channel-specific performance was assessed as the average balanced accuracy across LOO datasets.

#### 2.5.6 Visualizing cortical activation on a brain template

For data visualization purposes, statistical maps and ISCs were plotted on a template cortical surface. Briefly, AtlasViewer was used to spatially register the channels used in this study onto a common reference template (Colin27;Aasted et al., 2015;Collins et al., 1998). To do so, it uses anchor-based registration (Tsuzuki et al., 2007,^2012^), which uses a set of canonical landmarks from the 10-20 system from EEG (e.g., nasion, left, and right preauricular), in addition to another anchor point on the scalp to derive the affine that enables the translation from channel space to brain space. Once translated, Monte Carlo simulation is used to model the expected flight path of photons from a channel to the brain (Boas et al., 2002). These simulations account for the scattering and absorption properties of brain tissue, enabling a calculation of the sensitivity a channel has for recording from a given region. The underlying brain regions are then inferred from this spatial sensitivity profile (seeAbdalmalak et al., 2022for a plot showing the sensitivity profile obtained with the probe used in this study). These regions were then labeled using the Automated Anatomical Labeling (Tzourio-Mazoyer et al., 2002) which was independently validated by the Harvard Oxford cortical atlas (Desikan et al., 2006;Frazier et al., 2005;Goldstein et al., 2007;Makris et al., 2006).

## 3 Results

### 3.1 Group results

As shown inFigure 2,*Intact*conditions showed widespread significant ISCs.*BYD*showed significant ISCs in the superior, middle, and inferior frontal gyri, bilateral supramarginal and parietal lobule, the parieto-occipital junction, and superior and middle temporal lobes bilaterally. As expected, significant ISCs in the audio-only*Taken*condition were less diffuse compared to*BYD*but engaged several frontal and parietal regions such as the bilateral middle frontal gyrus, bilateral supramarginal gyrus, and bilateral inferior frontal gyrus as well as in temporal regions such as the bilateral middle temporal gyrus and right superior temporal gyrus. In contrast, both*Scrambled*conditions showed focal ISCs within the left temporal cortex, proximal regions of the inferior frontal gyrus and parieto-occipital junction. See theTable 1S-4Sin the Supplementary Materials for a complete list of significant regions.

**Fig. 2.**
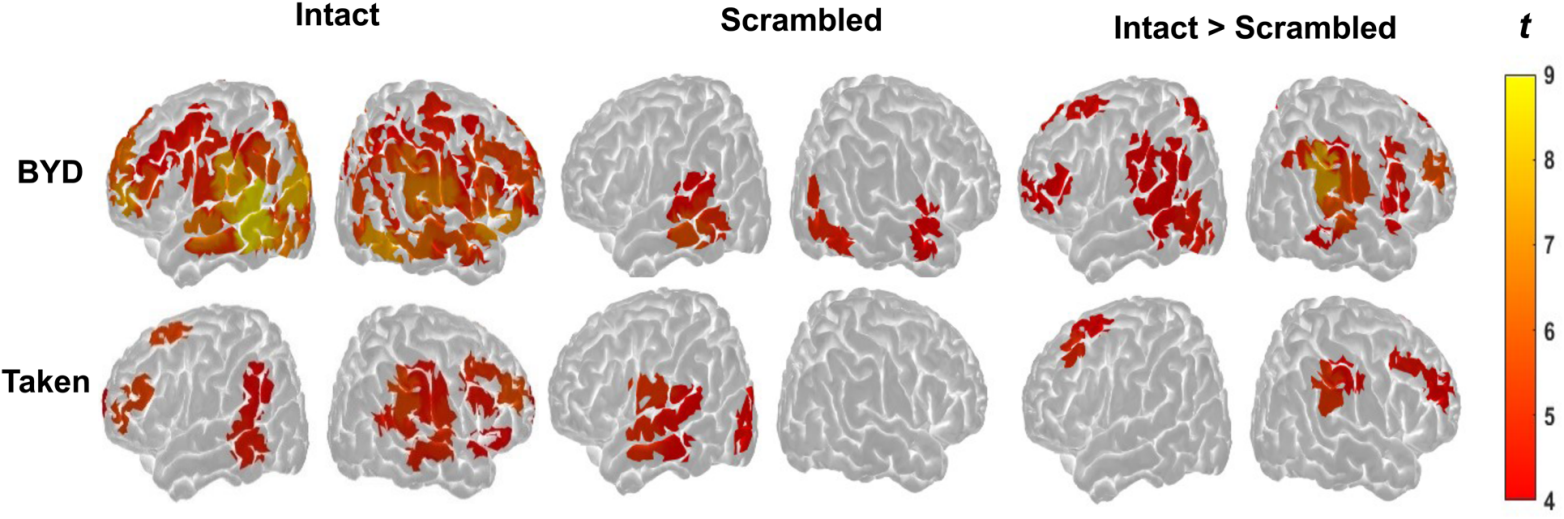
Significant group-level ISCs for*BYD*(top) and*Taken*(bottom) condition, for*Intact*,*Scrambled*, and*Intact*>*Scrambled*comparisons. The colormap denotes the strength of the*t*-score ranging from 4 (red) to 9 (yellow). The correspondence between the channel and the area of the brain was estimated using Monte Carlo Simulation and AtlasViewer (Aasted et al., 2015).

When*Intact*and*Scrambled*conditions were compared, a clear pattern of robust ISC emerged in*Intact*conditions compared to*Scrambled*conditions (seeFig. 2). In*BYD>BYD Scrambled*, peak frontal and parietal ISC was observed in the bilateral supramarginal gyri, inferior frontal gyrus, left superior and inferior parietal lobule, the left superior frontal gyrus, and bilateral middle frontal gyri. In the*Taken*>*Taken Scrambled*comparison, peak frontal and parietal ISC was observed in the right supramarginal gyrus, left anterior cingulate gyrus, bilateral middle frontal gyri, and pars triangularis. For all significant regions for each comparison, refer toTables 1&2.

**Table 1.** Significant regions in the*BYD*>*BYD Scrambled*comparison.

Channel name	*q*	*t* (25)	Channel name	*q*	*t* (25)
Right supramarginal gyrus	0	5.342	Left middle frontal gyrus	0.014	4.169
Right supramarginal gyrus	0	7.523	Left postcentral gyrus	0.023	4.049
Right superior temporal gyrus	0	5.747	Right middle temporal gyrus	0.023	4.063
Right middle frontal gyrus	0	5.690	Left supramarginal gyrus	0.023	4.051
Right angular gyrus	0.002	4.646	Left middle frontal gyrus	0.025	4.010
Left middle occipital gyrus	0.004	4.478	Right supramarginal gyrus	0.028	3.965
Right superior temporal gyrus	0.004	4.516	Left superior frontal gyrus	0.039	3.858
Right pars opercularis	0.005	4.449	Left inferior parietal lobule	0.04	3.839
Left middle temporal gyrus	0.005	4.412	Right pars opercularis	0.044	3.798
Right middle cingulate gyrus	0.007	4.307	Left middle temporal gyrus	0.047	3.747
Left superior parietal lobule	0.009	4.253	Left superior frontal gyrus	0.048	3.711

**Table 2.** Significant regions in the*Taken*>*Taken Scrambled*comparison.

Channel name	*t* (25)	*q*
Right supramarginal gyrus	5.110	0.003
Left anterior cingulate gyrus	4.807	0.004
Right pars triangularis	4.319	0.010
Right supramarginal gyrus	4.125	0.020
Right middle frontal gyrus	4.085	0.024
Left middle frontal gyrus	3.848	0.047

### 3.2 Associating suspense ratings with group-level changes in HbO and HbR activity

Previously published suspense ratings for the two narrative stimuli were used to further link neural synchronization across participants to a shared experience of the movie narratives (Laforge et al., 2020;Naci et al., 2014). As shown inFigure 3, HbO and HbR activity during*BYD*was significantly predicted by suspense ratings in the left inferior parietal lobule (HbO:*t*(234) = 3.293,*q*< 0.05; HbR*t*(234) = -3.564,*q*< 0.05), left postcentral (HbO:*t*(234) = 4.899,*q*< 0.001; HbR*t*(234) = -5.075,*q*< 0.001), and bilateral pars triangularis (right: HbO:*t*(234) = 3.069,*q*< 0.05; HbR:*t*(234) = -3.169,*q*< 0.05; left: HbO:*t*(234) = 3.412,*q*< 0.05; HbR:*t*(234) = -3.557,*q*< 0.05). For*Taken,*HbO and HbR channels were significantly predicted by suspense ratings in the right medial superior frontal gyrus (HbO:*t*(142) = 3.456,*q*< 0.05; HbR*t*(142) = -3.085,*q*< 0.05), left superior frontal gyrus (HbO:*t*(142) = 3.121,*q*< 0.05; HbR*t*(142) = -3.920,*q*< 0.05), right superior frontal gyrus (HbO:*t*(142) = 3.928,*q*< 0.05; HbR*t*(142) = -3.181,*q*< 0.01), and left middle occipital gyrus (HbO:*t*(142) = 3.769,*q*< 0.05; HbR*t*(142) = -4.161,*q*< 0.01). Notably, some of these significant regions overlapped with regions found in the group results, such as the left inferior parietal lobule and left postcentral gyrus in*BYD*and the left superior frontal gyrus in*Taken*, providing further evidence that the observed neural differences between conditions are due to higher-level processing of the movie narratives.

**Fig. 3.**
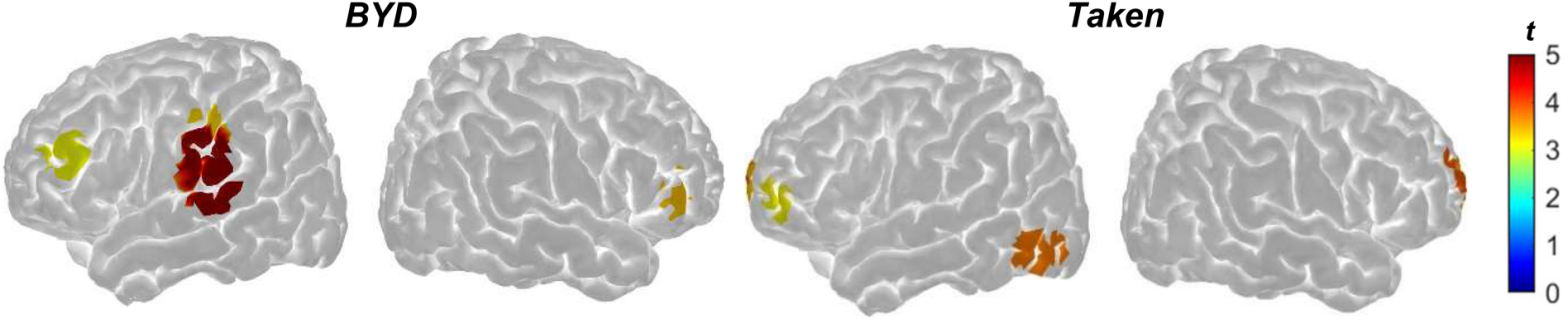
T-scores from the group averaged HbO activity (left and right sagittal view) depicting regions that were significantly predicted by suspense ratings (*q*< 0.05) in the*BYD*(left) and*Taken*(right) conditions after controlling for their*Scrambled*conditions. HbR (not shown) showed similar findings, but in the opposite direction. The colormap denotes the strength of the*t*-scores, with darker red colors indicating significant positive*t*-scores.

### 3.3 Assessing the consistency of ISCs

As expected, the normalized dot products in the*Intact*conditions were significantly larger than the*Scrambled*condition. Specifically,*BYD*had a significantly larger normalized dot product (*M*= 0.027,*SD*= 0.019) than*BYD Scrambled*(*M*= 0.011,*SD*= 0.009,*t*(25) = 4.447,*p*< 0.001) and*Taken*had a significantly larger normalized dot product (*M*= 0.017,*SD*= 0.014) than*Taken Scrambled*(*M*= 0.003,*SD*= 0.010,*t*(25) = 4.883,*p*< 0.001). Critically, when an individual participant’s normalized dot product was compared to what was obtained by chance, it was revealed that 24 out of 26 participants in*BYD*and 20 out of 26 in*Taken*were significantly different from chance (seeFig. 4for each participant’s consistency results). Specifically, in the*BYD*condition, the normalized dot products of participants 7 of -0.015 ($p_{unc}$= 1), 8 of 0.015 ($p_{unc}$= 0.120) did not differ from chance. Whereas in the*Taken*condition, the normalized dot products of participants 6 of 0.002, ($p_{unc}$= 0.198) 7 of -0.004 ($p_{unc}$= 0.992), 8 of 0.005 ($p_{unc}$= 0.006), 16 of -0.015 ($p_{unc}$= 1), 19 of -0.007 ($p_{unc}$= 0.999) and 26 of 0.003 ($p_{unc}$= 0.023) could not be distinguished from chance.

**Fig. 4.**
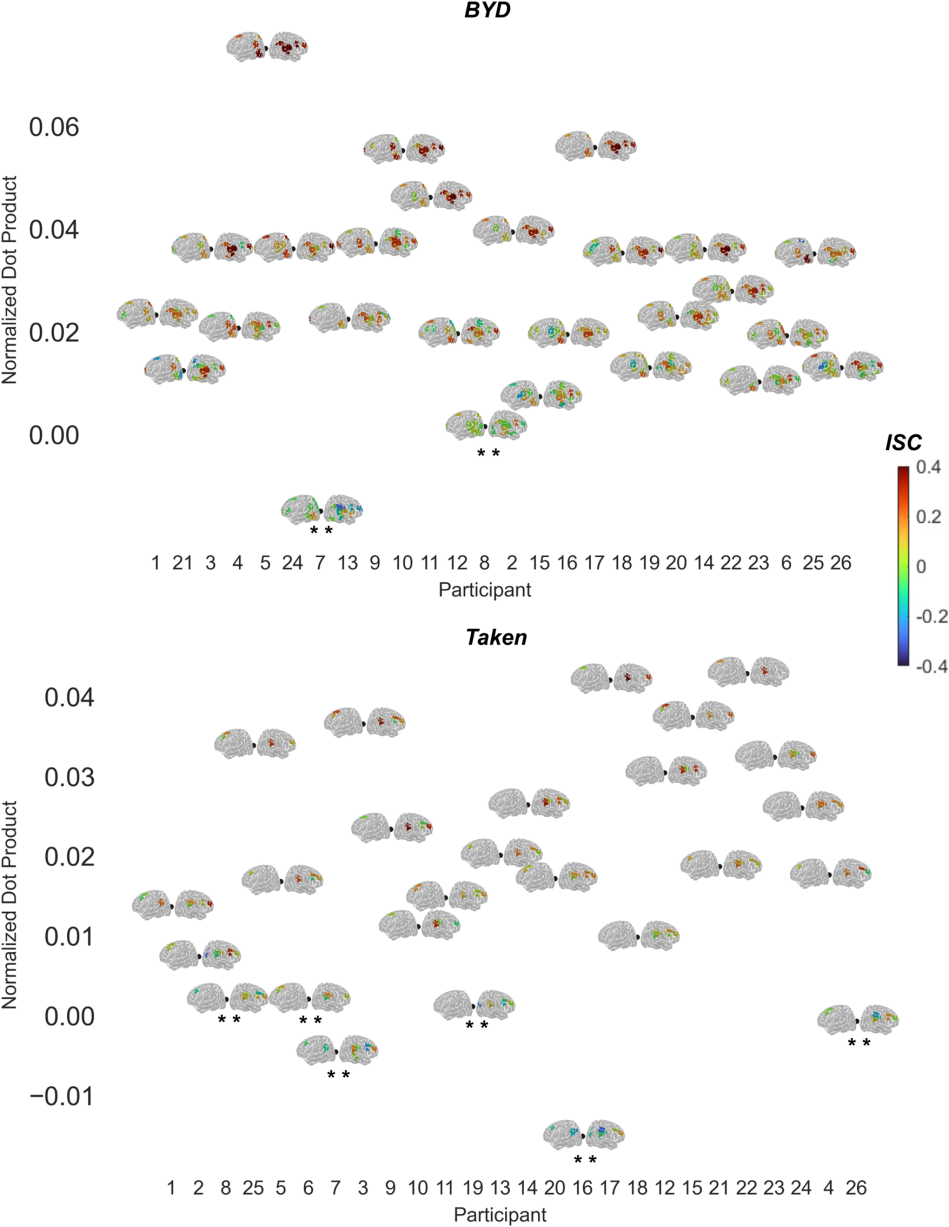
Normalized dot product values for the*BYD*(top) and*Taken*(bottom) condition. Participants are shown on the x-axis and normalized dot product on the y-axis. Asterisks indicate participants whose normalized dot product did not significantly differ from chance. The left and right lateral views of the brain show a given participant’s ISCs masked by channels that have significantly larger ISCs in the*BYD*>*BYD Scrambled*(top) or*Taken*vs.*Taken Scrambled*(bottom) in their respective LOO dataset, where the scale ranges from blue (-0.4) to red (0.4). Abbreviations: ISC = Inter-subject correlation; LOO = Leave-one out.

### 3.4 Assessing the sensitivity of ISCs

#### 3.4.1 BYD vs. BYD scrambled classification

The voting machine-learning approach could decode between*BYD*and*BYD Scrambled*with a balanced accuracy of 0.808 (*p*< 0.001). Specifically, the approach could distinguish the*BYD*condition from*BYD Scrambled*in 17 out of 26 participants and*BYD Scrambled*from*BYD*in 25 out of 26 participants (*recall*= 0.654,*p*= 0.029;*precision*= 0.944,*p*< 0.001). Additional details can be found inFigure 5Aand5B. Exploratory analyses revealed that several individual channels could sensitively decode between these conditions, including the right superior frontal gyrus and the right supramarginal gyrus which achieved the highest balanced accuracy scores (0.79 and 0.83, respectively) (seeFig. 5Cfor all channels). Notably, the performance of most classifiers was similar, suggesting that the results did not rely on complex non-linear relationships between the classifiers (details about the individual performance of classifiers can be found inFigure 2Sin the Supplementary Materials).

**Fig. 5.**
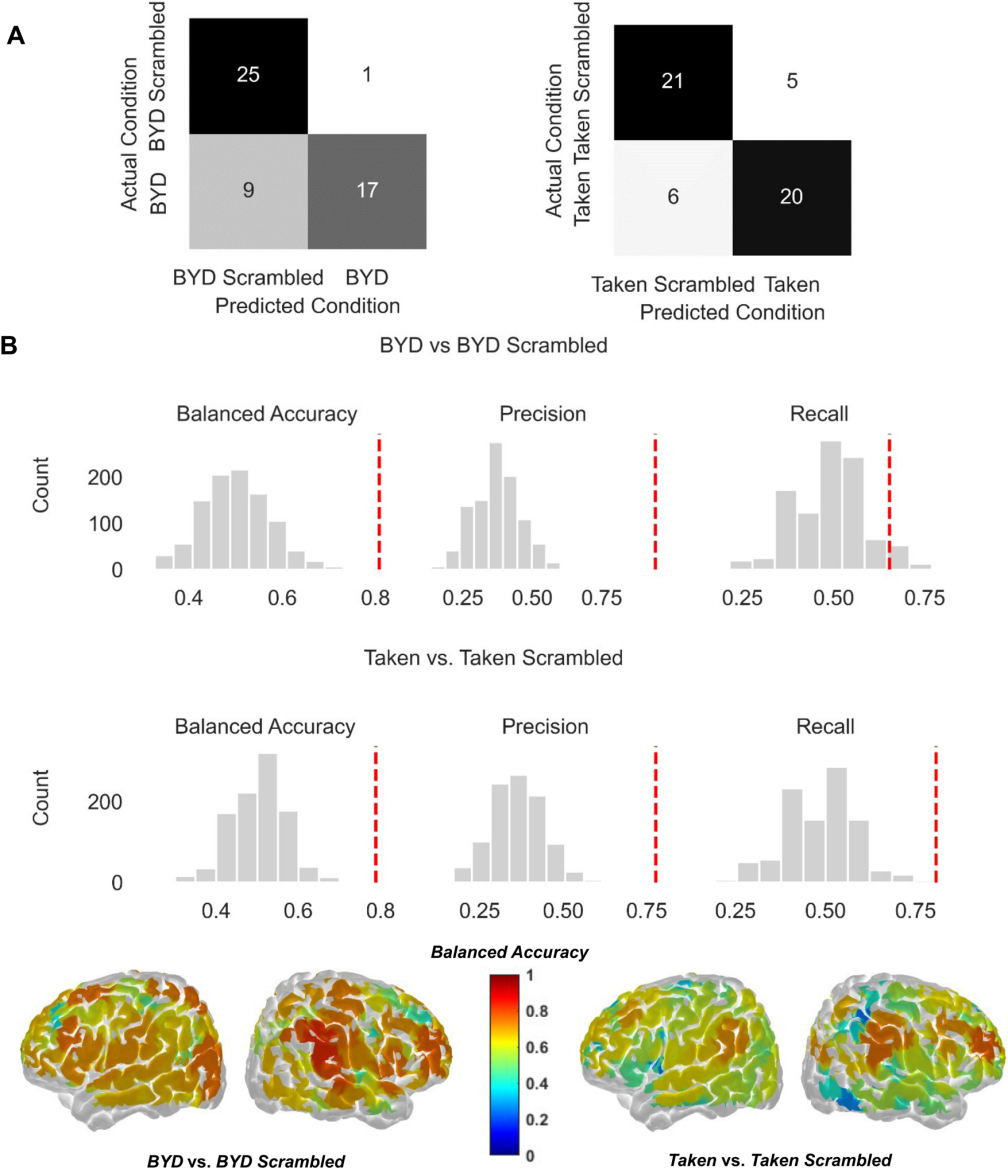
(A) Confusion matrices show the correct and incorrect predictions of the machine-learning approach. Participants are sorted by the predicted condition (x-axis) and actual condition (y-axis). (B) Histograms of performance metrics for*BYD*vs.*BYD Scrambled*(top) and*Taken*vs.*Taken Scrambled*(bottom). The histograms show the null distribution of balanced accuracy (left), precision (middle), and recall (right) that could be obtained by conducting the machine-learning approach on shuffled condition labels. The dashed red line reflects the actual performance metric obtained. (C) Channel decoding performance was determined by rerunning the machine-learning approach on individual channels. The brain maps show group balanced accuracy plotted on the whole brain (left and right lateral view), where the left indicates channels useful in distinguishing*BYD*vs.*BYD Scrambled*and the right channels useful in distinguishing*Taken*vs.*Taken Scrambled*. Darker red colors indicate higher balanced accuracy scores, whereas dark blue values indicate poor balanced accuracy.

#### 3.4.2 Taken vs. taken scrambled classification

The voting machine-learning approach could decode*Taken*and*Taken Scrambled*with a balanced accuracy of 0.788 (*p*< 0.001). Specifically, the approach could distinguish the*Taken*condition from*Taken Scrambled*in 20 out of 26 participants and*Taken Scrambled*from*Taken*in 21 out of 26 participants (*recall*= 0.769,*p*= 0.003;*precision*= 0.800,*p*< 0.001). Additional details can be found inFigure 5Aand5B. These results are likely driven by several channels, including the right middle frontal gyrus and, like*BYD*, the right supramarginal gyrus, which individually obtained balanced accuracy scores of 0.750 and 0.770 (seeFig. 5Cfor more details). As with the*BYD*vs.*BYD Scrambled*classification, most of the individual classifiers performed similarly well (seeFig. 3Sin the Supplementary Materials).

## 4 Discussion

This study aimed to determine whether the neural correlates of movie narratives were replicable and reproducible using fNIRS. First, the group analyses revealed that frontal and parietal regions showed higher ISC in*Intact*conditions compared to*Scrambled*conditions, replicating previous fMRI and EEG findings (Laforge et al., 2020;Naci et al., 2014,^2017^). Moreover, independently acquired ratings of suspense could be used to predict neural activity in a subset of these regions, thus further establishing the link between activity in these regions and the processing of higher-order components of the narratives. Critically, the findings of this study suggest that fNIRS is reproducible at the single-participant level. Participant-specific ISCs in regions that differed between*Intact*and*Scrambled*conditions were shown to be similar to the group. Namely, 24 out of 26 participants in*BYD*and 20 out of 26 in*Taken*had a normalized dot product greater than what was expected by chance. Finally, ISCs could be used to decode between*Intact*and*Scrambled*conditions with balanced accuracy ranging from 79–81%. These results support that fNIRS can robustly and reliably detect the neural correlates of movie narratives.

The significant ISCs in frontal and parietal regions during movie watching and listening overlap with areas previously identified in fMRI and EEG studies (Gao et al., 2020;Golland et al., 2007;Hasson et al., 2004;Laforge et al., 2020;Naci et al., 2014). This included the dorsolateral prefrontal cortex, inferior frontal and temporoparietal junction, which are key nodes of the frontoparietal and ventral attention networks (Corbetta & Shulman, 2002;Smith et al., 2009;Vossel et al., 2014). These results are in line with previous fNIRS studies, which have implicated the dorsal and prefrontal cortex in processing narrative stimuli (Mizrahi & Axelrod, 2023;Rowland et al., 2018;Somech et al., 2022). Importantly, these regions were absent in*Scrambled*conditions, which showed ISC primarily in temporal cortices, known for its processing of simple auditory information, and previously observed in fNIRS research (e.g.,Fishell et al., 2019;Luke et al., 2021). While there is broad overlap between our results and previous fMRI studies, the results of this study tend to be more distributed across the cortex, which likely reflects the reduced spatial resolution of fNIRS compared to fMRI.

These results support the broad use of movie narratives and fNIRS in clinical environments and naturalistic research settings. Notably, fNIRS has the advantage of being administered directly at a patient’s bedside, enabling measurement of both neonatal (Mitra et al., 2020;Tang et al., 2024) and adult populations (Bicciato et al., 2022) within the intensive care unit. In addition, fNIRS has been used to measure neural activity in patients with neurodegenerative diseases in scenarios where fMRI is impractical or potentially detrimental, including amyotrophic lateral sclerosis (Kuruvilla et al., 2013), Alzheimer’s disease (Blum et al., 2022;Keles et al., 2022), and in chronic disorders of consciousness patients (Abdalmalak et al., 2020). Future studies should consider having these patients watch or listen to movie narratives, as it enables the further exploration of higher-order cognitive processes with minimal time and effort. Moreover, movie narratives have been established as useful substitutes for resting-state procedures in developmental populations (Raschle et al., 2009;Vanderwal et al., 2015), which are an ideal population for fNIRS due to its robustness to motion (Pinti et al., 2020;Scholkmann et al., 2014). For these reasons, future studies should consider using movie narratives in place of resting-state procedures where appropriate.

The machine-learning approach used in this study aggregated 17 classifiers to predict which movie stimuli a participant was experiencing. It has been previously shown that prediction accuracy can vary dramatically based on which machine-learning classifier is used (e.g.,Naseer et al., 2016), as well as influenced by factors such as small sample size and number of training examples, which is a huge consideration in this study (Arbabshirani et al., 2017;Maier-Hein et al., 2018;Poldrack et al., 2020). Our methodology capitalizes on the multitude of available machine-learning approaches to produce a generalizable and robust estimate of the relationship between ISC and movie stimuli. While classifier performance is largely statistically similar, Bayesian, support vector classification, semi-supervised and quadratic discriminant classifiers performed best, whereas decision trees performed numerically worse (seeFig. 2S & 3Sin the Supplementary Materials). While choosing a single classifier could yield slightly better performance on this sample, the combination of all classifiers appears to provide an honest estimate of generalization error rather than cherry-picking a specific one. Indeed, with problems such as data leakage and the inherent empirical approach of machine learning, it is becoming even more important to produce honest estimates of classification performance (Lemm et al., 2011;Maier-Hein et al., 2018;Poldrack et al., 2020).

Overall, these results demonstrate that ISCs observed during movie watching and listening are consistent and sensitive at the individual level. Several explanations exist for the consistency observed in this study. From a technical perspective, using short channels to remove extra-cortical contaminations from the fNIRS signals and correcting motion artifacts reduce two of the main fNIRS confounding factors that can decrease reproducibility (Abdalmalak et al., 2022;Dravida et al., 2017;Kakimoto et al., 2009;Kirilina et al., 2012;Novi, Forero, et al., 2020,Novi, Roberts, et al., 2020;Wiggins et al., 2016;Zhang et al., 2011). Combining appropriate preprocessing with wide probe coverage and rich data analytic algorithms is likely key to achieving the observed high reproducibility. Indeed, these implementations and analyses help expand upon another study showing reliable single-participant reproducibility can be achieved with movie narratives and fNIRS (Mizrahi & Axelrod, 2023).

Despite these technical improvements, the neural results of some participants deviated from the group. It is unknown whether this is a consequence of the quality of the recording itself, a limitation due to the spatial sensitivity of fNIRS, or reflects differences in the way participants engaged with the clips. While the latter two cases cannot be ruled out, an exploratory analysis of several different quality indexes (e.g., SNR, coefficient of variation, scalp coupling index) was conducted, which found that only the coefficient of variation was significantly negatively related to consistency scores (see*Investigating the Relationship Between Signal Quality and Inter-Participant Variability*section in theSupplementary Materials). Future studies should map individual-specific cognitive responses and behaviors during the viewing of narrative stimuli to better understand the source of this variability.

### 4.1 Limitations

While the present study establishes the use of fNIRS to assess the neural correlates of narrative stimuli at the individual level, several limitations exist. One limitation is the lack of an independently acquired dataset, which poses challenges to accurately estimate inter-participant reproducibility. However, several steps were included (e.g., permutation testing) to reduce effects due to idiosyncrasies within the dataset. Second, one issue with*BYD*and*Taken*was that participants’ comprehension and other behavioral measures (e.g., engagement) of the movie clips were not assessed. Therefore, it is possible that some participants did not pay enough attention to the movies to recruit higher-order cognitive processes, which could potentially lead to false negatives. Third, fNIRS, unlike fMRI, only measures surface regions of the cortex. This limitation precludes measurement of posterior midline regions, such as the precuneus, which has been shown to be involved in the processing of movie narratives and naturalistic stimuli more broadly (Bottenhorn et al., 2018;Nastase et al., 2021). For this reason, future fNIRS research may lack the ability to investigate certain aspects of naturalistic stimuli which arise from these regions. Fourth, due to possibility of knowledge of the plot carrying over from*Intact*conditions to*Scrambled*conditions,*Scrambled*conditions always preceded their*Intact*counterpart. This makes it possible that our results are partially due to an order effect. While an order effect cannot be fully ruled out, explaining our results in terms of an order effect falls short in addressing how stimulus presentation order selectively produces significant ISCs in frontal and parietal regions in the*Intact*conditions, which have also been found in previous studies (e.g.,Gao et al., 2020;Hasson et al., 2004,^2010^). In addition, changes to the data that may drive an order effect (e.g., signal quality) would be partially controlled for by permutation testing. Finally, no channels were removed from the dataset. This was done to ensure that the estimates of inter-participant reproducibility were not biased by including only high-quality channels. Of note is that the quality of the channels should vary across participants but not across stimuli since the fNIRS cap was not removed, and all stimuli were perceptually comparable. Future studies should directly look at the impact of the inclusion of good and poor channel quality, particularly in regions that distinguish*Intact*and*Scrambled*conditions.

### 4.2 Conclusion

As shown in this study, the neural correlates of movie narratives, as acquired with fNIRS, are sufficiently reliable to detect higher-order cognitive processing in healthy participants. These findings support translating this paradigm to populations in which assessing higher-order cognitive capacities is difficult. Nevertheless, perfect inter-participant reproducibility was not achieved. Continued advancements in signal processing, multi-modal imaging, feature extraction, and task development can continue to clarify the underlying precision of fNIRS recordings as well as elucidate the underlying higher-order cognitive process under study (Kazazian et al., 2021;Naseer & Hong, 2015;Pinti et al., 2020;Yücel et al., 2021).

## Supplementary Material

Supplementary Material

## Data Availability

The code detailed in the method can be found here:https://github.com/TheOwenLab/fNIRS-Narrative-Stimuli. The data supporting the findings of this research are available on request to the corresponding author, pending a formal data-sharing agreement and approval from the local ethics committee.
