# Supplementary material for: Assessing the consistency and sensitivity of the neural correlates of narrative stimuli using functional near-infrared spectroscopy
Source: Imaging Neurosci (Camb). 2024 Oct 24;2:imag-2-00331. doi: 10.1162/imag_a_00331 (PMC12290713; doi:10.1162/imag_a_00331)

Validation of inter-subject correlation analysis using resting state data

To ensure that our data analysis and preprocessing used to extract ISCs with fNIRS was unlikely to be compromised by factors such as systemic physiological noise, the same analysis pipeline was applied in an independently collected sample of ten participants who participated in a twelve-minute resting-state task (see Abdalmalak *et al.*, 2022 for more details). The resting-state data were acquired with the same probe and fNIRS system. In the present work, it is assumed that the ISCs originated from the shared processing induced by the stimuli. However, since this coupling factor is absent in resting-state data acquired independently for each participant, there should be no correlation across participants, making the resting-state dataset ideal for validating our methodology.

The original twelve minutes of resting state were cut to 6 minutes to better match the duration of the movie clips. While canonical resting state networks exist, it is presumed that the temporality of these networks are not consistent across participants. As in fMRI (e.g., Naci *et al.,* 2014), this finding was confirmed, with no regions showing significant activation (see Figure 1S).

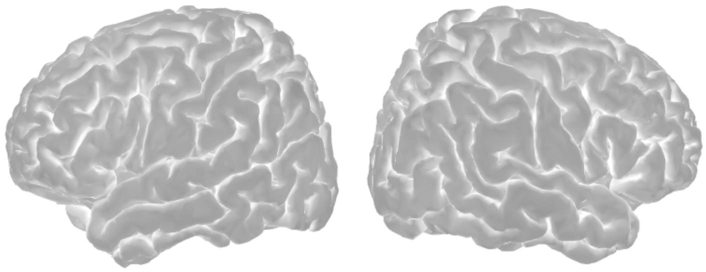

Figure 1S: Group level results (left and right sagittal view) depicting significant ISCs ($q < .05$). The data was collected from ten participants as they participated in twelve minutes resting state task (see Abdalmalak *et al.*, 2022 for more details). The twelve minutes of resting state were cut to 6 minutes in order to better match the task duration of the movie clips, but otherwise, preprocessing was identical to what is described in the methods of the main manuscript (see **Preprocessing**). No channels were significant after correction using the max *t* approach (Nichols & Holmes, 2002).

Table 1S: Significant regions in the *BYD* condition

Channel Name	*q*	*Z*	Channel Name	*q*	*Z*
Left Superior Frontal Gyrus	0	5.801	Left Middle Temporal Gyrus	0.002	5.041
Left Supramarginal Gyrus	0	5.792	Right Middle Temporal Gyrus	0.005	4.905
Left Middle Temporal Gyrus	0	8.636	Right Middle Cingulate Gyrus	0.005	4.932
Left Superior Temporal Gyrus	0	5.789	Right Supramarginal Gyrus	0.006	4.718
Left Middle Temporal Gyrus	0	8.03	Superior Parietal Lobule	0.006	4.77
Left Middle Occipital Gyrus	0	6.716	Left Precentral Gyrus	0.006	4.694
Left Middle Occipital Gyrus	0	6.192	Left Postcentral Gyrus	0.006	4.748
Left Middle Occipital Gyrus	0	7.86	Right Superior Temporal Gyrus	0.006	4.756
Right Pars Orbitalis	0	7.041	Left Pars Operculum	0.006	4.702
Left Middle Temporal Gyrus	0	6.508	Right Superior Temporal Gyrus	0.008	4.654
Right Superior Temporal Gyrus	0	5.792	Right Middle Frontal Gyrus	0.009	4.54
Right Pars Opercularis	0	5.593	Right Precentral Gyrus	0.009	4.59
Right Supramarginal Gyrus	0	6.323	Left Superior Temporal Gyrus	0.009	4.622
Right Superior Temporal Gyrus	0	5.74	Right Middle Temporal Gyrus	0.009	4.583
Left Superior Frontal Gyrus	0	7.29	Left Precentral Gyrus	0.009	4.577
Right Middle Temporal Gyrus	0	6.294	Right Postcentral Gyrus	0.012	4.471
Right Inferior Temporal Gyrus	0	7.339	Left Precentral Gyrus	0.015	4.386
Left Superior Frontal Gyrus	0	6.483	Right Superior Frontal Gyrus	0.016	4.341
Left Medial Superior Frontal Gyrus	0	5.81	Left Postcentral Gyrus	0.016	4.339
Left Medial Superior Frontal Gyrus	0	5.914	Right Pars Triangularis	0.017	4.308
Right Superior Temporal Gyrus	0	6.753	Right Precentral Gyrus	0.022	4.248
Left Postcentral Gyrus	0	7.509	Left Middle Frontal Gyrus	0.022	4.252
Right Supramarginal Gyrus	0.001	5.554	Right Inferior Parietal Lobule	0.025	4.187
Right Pars Opercularis	0.001	5.492	Right Middle Temporal Gyrus	0.025	4.178
Right Pars Triangularis	0.001	5.365	Left Middle Frontal Gyrus	0.026	4.159
Right Middle Frontal Gyrus	0.001	5.552	Left Cuneus	0.029	4.101
Right Middle Occipital Gyrus	0.001	5.459	Right Rolandic Operculum	0.029	4.071
Right Middle Occipital Gyrus	0.001	5.365	Right Superior Parietal Lobule	0.029	4.084
Left Middle Temporal Gyrus	0.001	5.338	Left Superior Temporal Gyrus	0.029	4.077
Left Middle Frontal Gyrus	0.001	5.375	Left Inferior Parietal Lobule	0.029	4.120
Right Pars Opercularis	0.002	5.287	Right Angular Gyrus	0.032	4.008
Left Inferior Temporal Gyrus	0.002	5.303	Right Pars Triangularis	0.033	3.982
Left Rolandic Operculum	0.002	5.037	Left Insula	0.033	3.989

Right Superior Occipital Gyrus	0.002	5.230	Right Postcentral Gyrus	0.033	3.976
Left Middle Temporal Gyrus	0.002	5.034	Right Precentral Gyrus	0.036	3.937
Left Middle Occipital Gyrus	0.002	5.291	Left Inferior Parietal Lobule	0.04	3.902
Right Angular Gyrus	0.002	5.233	Left Insula	0.042	3.865
Right Middle Temporal Gyrus	0.002	5.309	Right Middle Frontal Gyrus	0.044	3.841
			Left Pars Triangularis	0.046	3.834

Table 2S: Significant regions in the *BYD Scrambled* condition

Channel Name	*q*	*Z*
Left Middle Temporal Gyrus	0	5.733
Right Middle Occipital Gyrus	0.001	4.790
Left Middle Temporal Gyrus	0.001	4.879
Right Inferior Temporal Gyrus	0.006	4.502
Left Superior Temporal Gyrus	0.024	4.020
Left Superior Temporal Gyrus	0.031	3.933
Right Superior Temporal Gyrus	0.038	3.864

Table 3S: Significant regions in the *Taken* condition

Channel Name	*q*	*Z*	Channel Name	*q*	*Z*
Right Superior Temporal Gyrus	0	4.965	Right Pars Triangularis	0.002	4.470
Right Middle Frontal Gyrus	0	5.691	Right Pars Opercularis	0.002	4.436
Left Middle Temporal Gyrus	0	4.597	Right Superior Temporal Gyrus	0.005	4.385
Right Supramarginal Gyrus	0	5.533	Right Postcentral Gyrus	0.009	4.293
Right Superior Temporal Gyrus	0	4.751	Right Supramarginal Gyrus	0.01	4.267
Right Superior Temporal Gyrus	0	4.563	Left Middle Temporal Gyrus	0.013	4.208
Left Middle Frontal Gyrus	0	5.462	Right Pars Orbitalis	0.038	3.929
Right Pars Triangularis	0	5.016	Left Supramarginal Gyrus	0.044	3.893
Left Superior Frontal Gyrus	0	5.414	Right Medial Superior Frontal Gyrus	0.05	3.842
Left Pars Triangularis	0	5.443	Right Middle Temporal Gyrus	0.05	3.853

Table 4S: Significant regions in the *Taken Scrambled* condition

**Channel Name**	***q***	**Z**
**Left Superior Temporal Gyrus**	0.003	4.901
**Left Superior Temporal Gyrus**	0.018	4.252
**Left Middle Occipital Gyrus**	0.023	4.119
**Left Superior Temporal Gyrus**	0.037	3.879
**Left Middle Temporal Gyrus**	0.043	3.822

Table 5S: List of Models and Hyperparameters Optimized

Model	PCA	Hyperparameters
**Extra Trees Classifier**	**FALSE**	**n_estimators**
		**criterion**
		**max_depth**
		**min_samples_split**
		**min_samples_leaf**
		**max_features**
		**bootstrap**
		**class_weight**
**AdaBoost Classifier**	**FALSE**	**n_estimators**
		**learning_rate**
		**algorithm**
**Bagging Classifier**	**FALSE**	**n_estimators**
		**bootstrap**
		**bootstrap feature**
		**oob score**
**Ridge Regression**	**FALSE**	**alpha**
		**fit_intercept**
		**max_iter**
		**tol**
**Linear Regression**	**FALSE**	**C**
		**penalty**
		**solver**
		**max_iter**
**Label Progagation**	**TRUE**	**gamma**
		**max_iter**

		tol
Label Spreading	TRUE	gamma
		alpha
		max_iter
		tol
Linear Discriminant Analysis	TRUE	NONE
Quadratic Discriminant Analysis	TRUE	reg_param
Support Vector Machine	FALSE	C
		kernel
NuSupport Vector Machine	FALSE	nu
		kernel
		shrinking
		tol
Gaussian Process	FALSE	max_iter_predict
		n_restarts_optimizer
		kernel
Gaussian Naïve Bayes	FALSE	var_smoothing
Bernoulli Naïve Bayes	FALSE	alpha
		binarize
		fit_prior
K-Nearest Neighbors	FALSE	n_neighbors
		weights
		p
Nearest Centroid	FALSE	metric
		shrink_threshold
Extreme Gradient Boosted Trees	FALSE	learning_rate
		n_estimators
		max_depth

	**min_child_weight**
	**gamma**
	**subsample**
	**colsample_bytree**
	**colsample_bylevel**
	**colsample_bynode**
	**reg_alpha**
	**reg_lambda**
	**objective**

Table 6S: Motion Correction Information

Participant	Proportion of Samples Corrected via Spline Interpolation
1	0.005933502
2	0.004638348
3	0.006090724
4	0.005665631
5	0.012344009
6	0.016591081
7	0.014222664
8	0.014338059
9	0.01693252
10	0.011806191
11	0.005155104
12	0.012169285
13	0.019071334
14	0.014030142
15	0.018771129
16	0.013178176
17	0.012561153
18	0.008583732
19	0.016015886
20	0.014755143
21	0.019154988
22	0.016148783
23	0.01657002
24	0.020289953
25	0.013782739
26	0.020496418

Individual Classifier Results

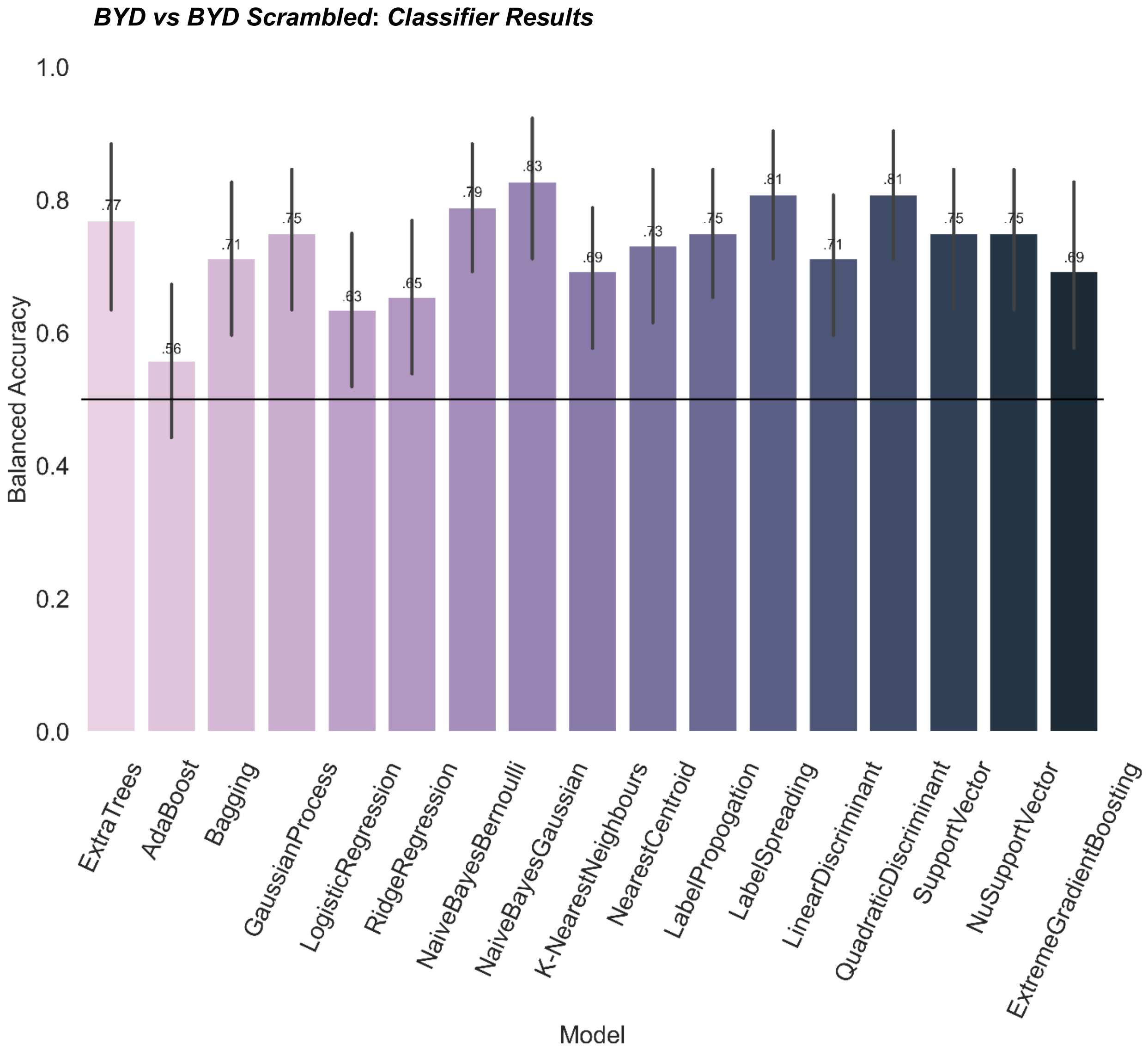

Supplementary Figure 2S: Bar plots of mean balanced accuracy scores for *BYD* vs *BYD Scrambled* for 17 different sci-kit learn classifiers (Pedregosa *et al.*, 2011). Vertical lines for each bar indicate the 95% confidence interval and the horizontal black line indicates chance.

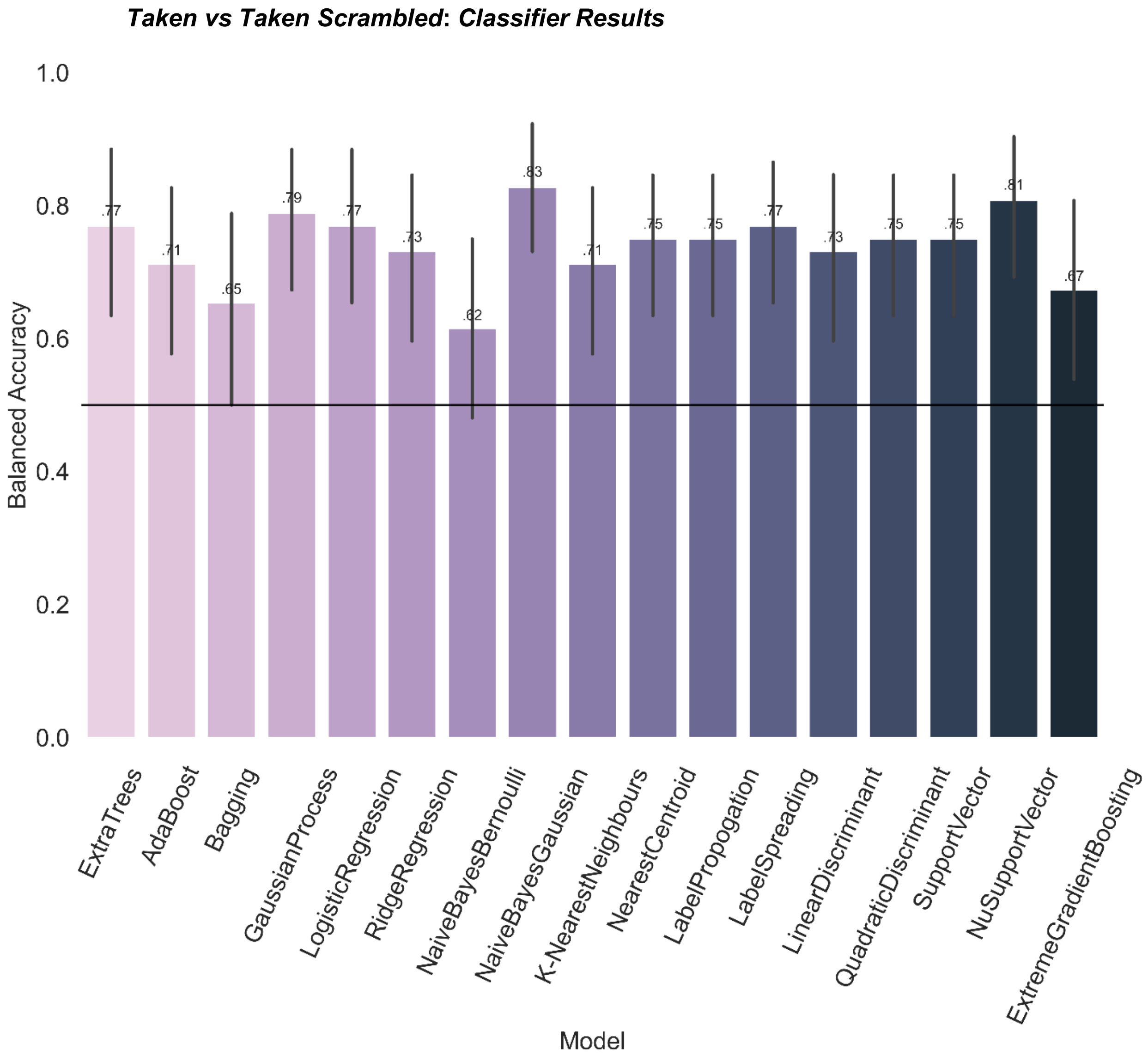

Supplementary Figure 3S: Bar plots of mean balanced accuracy scores for *Taken Scrambled* for 17 different sci-kit learn classifiers (Pedregosa *et al.*, 2011). Vertical lines for each bar indicate the 95% confidence interval and the horizontal black line indicates chance.

Investigating the relationship between signal quality and inter-participant variability

Generally, participants had largely similar ISC for the *Intact* conditions, but there were some exceptions. One potential cause for this deviation is the quality of underlying signal. To this end, the normalized dot products obtained from the consistency analysis were correlated with several other quality metrics used in the fNIRS literature. This includes channel-specific metrics such as scalp coupling index (the presence of heart beat in the signal; Pollonini *et al*., 2014), signal to noise ratio (μ/σ; SNR), coefficient of variation (σ/μ), and HbO and HbR anti-correlation (Cui *et al*., 2010). Where appropriate, these values were computed across conditions. Only channels that were significant in the *BYD* > *BYD Scrambled* comparison (when looking at the BYD consistency scores) and the *Taken* > *Taken Scrambled* comparison (when looking at the *Taken* consistency scores) were considered. For each channel-specific quality metric, the median was taken. Note that these quality metrics were calculated following bandpass filtering but prior to short channel regression in order to see which quality measures still influenced this consistency measure after the data cleaning procedure. In addition to these channel-specific quality measures, quality measures that related to the overall quality of a participant's data, such as the number of clean short channels and number of channels that met our quality cutoff (e.g., < 8 SNR) were used.

Interestingly, only the coefficient of variation showed a significantly negative correlation to the consistency scores for both HbO and HbR in *BYD* (HbO: $r(26) = -0.413$, $p = .036$; HbR: $r(26) = -0.403$, $p = .042$) and HbO in *Taken* (HbO: $r(26) = -0.458$, $p = .019$; HbR: $r(26) = -0.189$, $p = .356$). None of the other channel-specific or overall quality metrics showed any correlation with the consistency scores ($p > 0.065$). While this suggests that data quality plays some role in defining dissimilarity to the group there are likely other factors as well.

Exploring neural differences in higher-order cognitive processes between BYD and Taken

An interesting exploratory analysis is to attempt to identify neural differences in higher-order cognition between the *BYD* and *Taken* conditions, after considering ISCs present in their respective *Scrambled* condition. To this end, a two-tailed *t*-test comparing the *Intact* results from *BYD* (i.e., *BYD* > *BYD Scrambled)* to the *Intact* results from *Taken* (i.e., *Taken* > *Taken Scrambled*) was conducted. This *t*-score was compared to a two-tailed distribution of maximum and minimum *t*-scores, derived using the phase scrambling approach discussed in the main

manuscript. Two channels (the left precentral and left inferior parietal lobule) were significantly larger for *BYD* and one channel (the left middle frontal gyrus) was significantly smaller for *Taken*. While these regions are involved in higher order cognitive processing such as attention and language, these results are complicated by both the differences in sensory input (audio-visual versus audio-only) and the difficulties in quantifying the qualitative differences between the clips. Future studies can identify taking these concerns into consideration, will be better suited to identify the specific functions accomplished by these regions in the context of narrative stimuli.

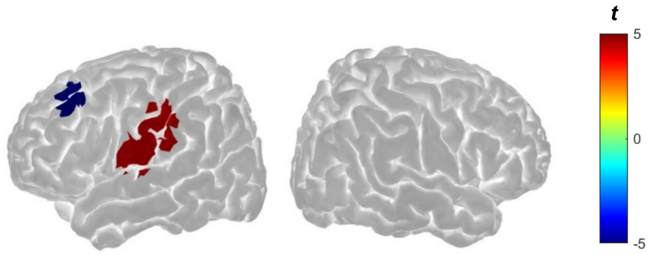

Supplement: Supplementary Material [file imag_a_00331-supp.pdf]
